# Middlesex Hospital Festival Dinner

**Published:** 1894-04-21

**Authors:** 


### The Institutional Workshop.

### MIDDLESEX HOSPITAL FESTIVAL DINNER.

The festival dinner in aid of the funds of the Middlesex Hospital, which was held on Friday evening at the Whitehall Rooms, Hotel Metropole, could not fail under the circumstances to be a conspicuous success. Not only did H.R.H. the Prince of Wales occupy the chair, but Her Majesty the Queen, through Sir Henry Ponsonby, sent from Florence a telegram of good wishes for the future of the institution. The company, too, which numbered some 220, was very representative, and included not a few of those whose self-sacrificing work in aid of the London charities is a matter of universal knowledge. Right and left respectively of the noble chairman were the Lord Chancellor and Lord Sandhurst, chairman of the Weekly Board of Governors; his Eminence Cardinal Vaughan, denoted by his presence the sympathy with and interest in metropolitan charitable work which Roman Catholicism has not failed to evince; the City was represented by the Lord Mayor, Aldermen and Sheriffs J. V. Moore and Dimsdale and Alderman Sir D. Evans, and the guilds by the Master of the Vintners' Company. Other prominent guests were the Earls of Onslow, Lathom, and Gosford, the Earl Waldegrave, the Marquis of Breadalbane, Lords Carrington and Amherst of Hackney; Admiral Sir E. Commerell representing the Navy; and General Lord Methuen and Major-Generals F. T. Lloyd and Ellis the Army. The Lower House of Parliament was represented by the Secretary of State for War, the Right Hon. H. Campbell Bannerman, Sir E. Lechmere, Sir F. Seager Hunt, Mr. W. Woodall, Mr. E. Boulnois, Mr. F. Wootton Isaacson, Mr. J. M. Paulton, Mr. T. P. O'Connor, and Mr. J. C. Macdona, and education by Mr. J. R. Diggle, while the drama supplied Messrs. S. B. Bancroft and S. Comyns Carr and Sir Augustus Harris, the first-named, as is well-known, being on the weekly board of the hospital. As might have been expected, there was a very large gathering of the governing body, including Mr. J. Bell Sedgwick, the deputy chairman, and of the medical profession, among which latter may at least be mentioned the two pillars of the hospital, Dr. W. Cayley, the senior physician, and Mr. J. W. Hulke, the senior surgeon. The festival being specially in aid of the funds for the erection of the proposed cancer wards, less reference perhaps than might have been expected was made to the actual work of the hospital. Incidental references by Lord Sandhurst bore testimony to the thoroughness with which the work of the hospital is carried on, to the introduction of such advantages as the electric light and the provision for the comfort of the nurses. The completion of the little chapel, it may be added, is also rapidly approaching, private assistance being solely responsible for this useful addition. The Middlesex Hospital is one which, in a lateral direction, is capable of a good deal of expansion, but like most institutions of a similar character, want of funds delays—or prevents such a desirable proceeding. The actual speech-making was short and to the point.

In giving "The Health of her Majesty the Queen," a toast which was drunk with much enthusiasm, his Royal Highness said he only wished to remind those present that the Queen had been patron of the Middlesex Hospital for over half-a-century, and had been a subscriber of 100 guineas ever since she came to the throne.

In a few brief but well-chosen phrases the Lord Chancellor proposed "The Prince and Princess of Wales and the other Members of Royal Family," pointing out the readiness of his Royal Highness to assist every good and charitable cause, and the thoroughness with which he interested himself in it when he thought it a good one. They all rejoiced to know that the Princess of Wales was regaining strength, and the more she came among the people the greater pleasure she gave. Of the other members of the Royal Family he would only say that they had shown the greatest desire to do all that they could to further the good of the people, and he might perhaps be permitted to allude to one—his Royal Highness the Duke of York—who, it was gratifying to see, was following in the footsteps of his Royal father, and was ready to give the same assistance to good works by every means in his power.

The Prince of Wales, in acknowledging the toast, which was very heartily received, evoked laughter and cheers by saying that that was certainly not the first time that he had presided at a public dinner, but that there was not one which had given him greater pleasure and satisfaction than that at which they had met together that night. Without a pause, his Royal Highness passed on to propose, "The Army, Navy, and Reserve Forces," expressing with (for him) unusual freedom his pleasure that her Majesty's Government had thought fit to increase the navy—a sentiment which was hailed with much enthusiasm. "In strengthening our navy," added the Prince, "God forbid that it should imply in any way that we wish to threaten other countries. Just the reverse. In order to be at peace we must be strong, and be assured our best policy is to strengthen our first line of defence—the navy. I hope that the motto of which our volunteers are so proud may be extended to the navy, that of 'Defence, not Defiance.'"

Admiral Commerell and Lord Methuen responded respectively for the navy and for the other branches of the service, the former relating an anecdote to show how the Duke of York, from a sense of duty, sacrificed a visit to Goodwood to the orders he had received to take his torpedo boat out to Spithead, and the latter bearing testimony to the improved *morale* of the army.

The Prince of Wales, who was again received with cheers, said: My Lords and Gentlemen,—Before proposing to you the toast of the evening, I have to rectify an omission. There is a telegram which Lord Sandhurst has received from the Queen, through Sir Henry Ponsonby. It is in these words:—"The Queen hopes that the Middlesex Hospital may continue to prosper." (Cheers.) I know, gentlemen, how gratified the Queen will be at the reception of this telegram, for I know what a great deal of interest she has taken in the welfare and prosperity of this excellent institution. As you are aware, gentlemen, the Middlesex Hospital is a very old one. It was founded in 1745 for sick and lame patients, and next year will celebrate its 150th anniversary. (Cheers.) In 1792 the first cancer ward was opened, and since that time ward after ward has been added to the hospital. I may mention that two years ago your income amounted to upwards of £20,000, but your outgoings were very large, upwards of £37,000, showing a deficit of between £16,000 and £17,000. It therefore proves to you how very necessary it is that we should support this excellent charity to the best of our ability. There have been many festival dinners, but I wish to say that I think, on this occasion, this is an especial festival dinner, because I wish to bring before you a scheme which I know lies very much at the heart of the governors, subscribers, and medical staff of this hospital. (Cheers.) As you are aware, there are these cancer wards, and the great object has been that in having them the patients will always remain "until relieved by art or released by death." That terrible disease is one we all know which has baffled up till now the endeavours of our cleverest physicians, and although we may alleviate it by operations for a time, it is, alas! difficult entirely to counteract. The governors have thought for some time that it would be possible to erect another building, and I am glad to think they have found a site for it. They will thereby be able to move to this new building those serious cases of cancer which are now in the present hospital. By so doing, also, they will be able to free these wards and put in the serious cases of lying-in patients—(hear, hear)—who through want of space have at the present time to be accommodated at their own homes by the medical staff. There is another point which I wish to bring to your notice. You know that there are young women who have arduous duties to perform—domestic servants, and girls in shops, who have to stand a long time and have to carry heavy weights, and to perform every kind of menial duty. Unfortunately they often incur at an early age serious ailments, which, if not cured at once, may prove fatal, or lead to the ruin of their health. (Hear, hear.) If these cases can be taken in hand by sending them to these wards, which we hope may be made, a great and philanthropic and good object will be carried out. At present it is very difficult to relieve these cases. They have to be attended to as best they can, but they cannot be looked after in the way we should wish. That is one of the reasons, therefore, why I wish to point out to you how essential it is that we should erect this new building and obtain these other wards to which I have alluded. The building will, I understand, cost £12,000, and I will mention that there are only 26 female cancer beds, while there are 200 applications, so that not even a third could be attended to. I may mention to you also another circumstance. Lord Sandhurst has furnished me with some statistics as to beds for cases peculiar to women. In the London Hospital there are 28 beds for such cases; in St. Bartholomew's, 28; in St. Thomas's, 22; in Guy's, 18; in Charing Cross, 13; in St. George's, 14; in St. Mary's, 13; in Westminster, 12; and in Middlesex, I am sorry to say, only eight. The donations that have been given at various times have been very large, and I must mention one of particular interest. One hundred and two years ago Sir Samuel Whitbread settled the interest of £4,000 upon this hospital. His great grandson, Mr. Samuel Whitbread, has given the handsome donation of £1,000. (Cheers.) From abroad also you have received various sums, Baron Hirsch having given £2,000. (Cheers.) You have before you a brief historical account of the origin of the hospital. Were I to go into all the statistics, it would only weary you and take me some considerable time to master the details. I appear before you to-night on account of a great, philanthropic object—the object being to maintain not only this existing most excellent and most valuable hospital, but to ask you to do your utmost to accomplish what is so much required, to give what you can towards the erection of this new building, which will prove such a boon to those unfortunate women who work and slave so hard. (Cheers.) I ask you to give your help to my noble friend Lord Sandhurst. I know full well the deep interest he takes in everything that concerns the institution, and I know also how much he is indebted to the medical staff—(cheers)—for the untiring energies they have displayed in their profession, and the assistance they have rendered to their patients. It would be invidious to mention them by name, but I may perhaps be permitted to allude to Mr. Hulke, the senior surgeon. (Cheers.) I congratulate him on having been elected to the high post of President of the Royal College of Surgeons. With no more words, I will now give "Prosperity to the Middlesex Hospital." (Cheers.)

Lord Sandhurst, who was loudly cheered, dealt more generally with the work of the hospital, in the longest speech of the evening. After acknowledging the warmth with which the toast had been received, and the powerful appeal made on behalf of the institution by the Prince of Wales, the Chairman of the Weekly Board said, amid applause, that on behalf of the governors he had telegraphed their grateful and respectful thanks to the Queen for her messsge. Their record for nearly 150 years had been an honourable one, and the present board would take care that the reputation of the hospital did not suffer, and that her Majesty's hope might not be falsified. Passing thence to business details, Lord Sandhurst said that at the hospital they could not do more than they were now doing in the in-patient line; paid a cordial and graceful tribute to the death-roll of the past year, and reviewed the changes in the composition of the board, and endorsed his Royal Highness's remarks with regard to the obligations under which the governing body were to the medical staff, at the same time making especial reference to Dr. Cayley. This indebtedness of the governors Lord Sandhurst extended to the secretarial staff. After expressing the solicitude of the Weekly Board for the welfare of the hospital servants, alluding to the progress being made with the construction of the chapel and the utility and extension of the Samaritan Fund, Lord Sandhurst concluded: "His Royal Highness has performed the double function of presiding at this dinner and also giving a tremendous lift to the voluntary system. Every day our circumstances become more painful, the competition of charities is something tremendous, and expense, with the march of science, naturally increases. The maintenance of the voluntary system depends entirely upon the way the hospitals are managed. If you can persuade the public that you have a deserving institution, that your patients are well looked after, and that the medical staff is first rate, I am persuaded you will not ask the public for money in vain. We have no philanthropic finance. We ask the public to trust us. If the public do not trust us you may depend upon it we are not worth trusting, and the sooner we go under the better, and make way for others. I am strongly in favour of the voluntary system, and cannot help thinking that it is in itself Christian, noble, and self-denying." This speech, which secured a most cordial reception, brought the proceedings to a close, but a list of subscriptions, to which the Prince of Wales contributed 100 guineas, was announced, the total amount reaching nearly £6,000.

As the organisation and regulation of sympathy and interest is no less important than the collection of subscriptions and donations—the two functions are not necessarily synonymous—it seems more than probable that if Lord Sandhurst could arrange, at the close of a function of this kind, for a conversazione at which the various and representative governors could meet, exchange views, extend knowledge, and bring about increasing solidarity, much might be done for the cause of the voluntary system. The hundred and fiftieth anniversary of the existence of the hospital which is now approaching seems to offer a special opportunity for something of the nature indicated. Let us hope it will be utilized.

